# Simultaneous Determination of Four Tanshinones by UPLC-TQ/MS and Their Pharmacokinetic Application after Administration of Single Ethanol Extract of Danshen Combined with Water Extract in Normal and Adenine-Induced Chronic Renal Failure Rats

**DOI:** 10.3390/molecules21121630

**Published:** 2016-11-28

**Authors:** Hong-Die Cai, Shu-Lan Su, Yonghui Li, Zhenhua Zhu, Jianming Guo, Yue Zhu, Sheng Guo, Dawei Qian, Jinao Duan

**Affiliations:** 1 Jiangsu Collaborative Innovation Center of Chinese Medicinal Resources Industrialization, National and Local Collaborative Engineering Center of Chinese Medicinal Resources Industrialization and Formulae Innovative Medicine, and Jiangsu Key Laboratory for High Technology Research of TCM Formulae, Nanjing University of Chinese Medicine, Nanjing 210023, China; caihongdie@foxmail.com (H.-D.C.); 04040416@163.com (Z.Z.); guojianming@163.com (J.G.); zhuyue@163.com (Y.Z.); gsh916@163.com (S.G.); qiandwnj@126.com (D.Q.); 2 Hainan Provincial Key Laboratory of R&D of Tropical Herbs, School of Pharmacy, Hainan Medical University, Haikou 571199, China; lyhssl@126.com

**Keywords:** *Salvia miltiorrhiza* Bge., tanshinones, pharmacokinetics, UPLC-TQ/MS, chronic renal failure

## Abstract

*Salvia miltiorrhiza*, one of the major traditional Chinese medicines, is commonly used and the main active ingredients—tanshinones—possess the ability to improve renal function. In this paper, the UPLC-TQ/MS method of simultaneously determining four tanshinones—tanshinone IIA, dihydrotanshinone I, tanshinone I, and cryptotanshinone—was established and applied to assess the pharmacokinetics in normal and chronic renal failure (CRF) rat plasma. The pharmacokinetics of tanshinones in rats were studied after separately intragastric administration of *Salvia miltiorrhiza* ethanol extract (SMEE) (0.65 g/kg), SMEE (0.65 g/kg) combined with *Salvia miltiorrhiza* water extract (SMWE) (1.55 g/kg). The results showed C_max_ and AUC_0–t_ of tanshinone IIA, tanshinone I, cryptotanshinone reduced by 50%~80% and CLz/F increased by 2~4 times (*p* < 0.05) in model group after administrated with SMEE. Nevertheless, after intragastric administration of a combination of SMWE and SMEE, the C_max_ and AUC_0–t_ of four tanshinones were upregulated and CLz/F was downregulated, which undulated similarity from the model group to the normal group with compatibility of SMEE and SMWE. These results hinted that SMWE could improve the bioavailability of tanshinones in CRF rats, which provides scientific information for further exploration the mechanism of the combination of SMWE and SMEE and offers a reference for clinical administration of *Salvia miltiorrhiza*.

## 1. Introduction

*Salvia miltiorrhiza*, a traditional Chinese herbal medicine, derived from the root of *Salvia miltiorrhiza* and belonging to the *Salvia* genus of family of Labiatae, has been used widely in clinics for the treatment of coronary heart diseases, particularly angina pectoris and myocardial infarction [1,2]. Previous research demonstrated that *Salvia miltiorrhiza* possessed various pharmacological effects for improving cerebral ischemia reperfusion injury, blood rheology, platelet function, anti-hypertensive, anti-inflammatory, and protecting the cardiovascular system [3,4,5,6,7].

Active ingredients in *Salvia miltiorrhiza* mainly include hydrosoluble salvianolic acids and liposoluble tanshinones. Pharmacological activities of salvianolic acids include anticancer activity on head and neck cancer and precancer cells [8], protection against polychlorinated biphenyls-induced oxidative stress [9], amelioration of central nervous system autoimmunity by suppressing Th1 responses [10], improvement of TNF-α induced cerebral microcirculatory changes in a micro-invasive mouse model [11], inhibition of endothelial dysfunction and vascular remodeling in spontaneously hypertensive rats [12], and so on. Tanshinones process anti-inflammatory activities in THP-1 macrophages [13], inhibit human esophageal cancer cell growth [14], suppress disruption of the blood-brain barrier [15], ameliorate bleomycin-induced pulmonary fibrosis [16], and prevent the loss of nigrostriatal dopaminergic neurons [17], and so on. Moreover, research shows *Salvia miltiorrhiza* extract, containing salvianolic acids and tanshinones, can prevent renal interstitial fibrosis in rats by regulating the expression of TSP-1, TGF-β1, VEGF [18]. Salvianolate can improve renal function and alleviate the progress of CRF by increasing renal blood flow, decreasing the renal oxygen consumption and improving energy metabolism in renal tissues [19]. Salvianolic acid B exhibited protection on renal interstitial fibrosis in rats induced by HgCl_2_ by increasing organism function against oxidative damage and alleviating lipid oxidation injury [20]. Tanshinone IIA significantly attenuated renal fibrosis in five out of six nephrectomized rats [21] and retarded glomerular sclerosis by enhancing the expression of nephrin and inhibiting the expression of TGF-β1 in kidney [22]. The large aggregation of studies demonstrated the pharmacokinetic profile of tanshinone IIA, cryptotanshinone, dihydrotanshinone I, and tanshinone I in normal rats with intragastric administration [23,24,25]. However, the biological environment in the body in a pathological state was different from its normal status, such as the variation of intestinal microecology in CRF Patients [26]. The pharmacokinetic profile after intragastric administration was closely associated with intestinal absorption. Therefore, it is necessary to explore the pharmacokinetic profile of tanshinones in CRF rats. Furthermore, studies showed the effective constituents in liposoluble or water extract of *Salvia miltiorrhiza* can interact in normal rats, changing pharmacokinetic parameters of various constituents [27,28,29,30]. Hence, the pharmacokinetic process of any single component cannot accurately represent the pharmacokinetic process of *Salvia miltiorrhiza*. Furthermore, the influence of water-soluble compounds on the pharmacokinetic profile of tanshinones in CRF rats was seldom reported. Therefore, multi-component pharmacokinetic research of SM would provide a reference for clinical applications in CRF.

The aim of the study is to establish a new UPLC-TQ/MS method based on the previous study method [31] and to explore and compare the difference of the pharmacokinetic profiles of tanshinones in normal and CRF rats and evaluate the influence of water extract of *Salvia miltiorrhiza* on the pharmacokinetic parameters of tanshinones in normal and CRF rats, providing a reference for clinical administration of *Salvia miltiorrhiza*.

## 2. Results and Discussion

### 2.1. Components Analysis of SMEE and SMWE

The constituents in SMEE were mainly tanshinones, including tanshinone IIA, cryptotanshinone, dihydrotanshinone I, tanshinone I, with the contents of 12.63 mg/g, 43.73 mg/g, 10.27 mg/g, and 6.25 mg/g, respectively. While the constituents in SMWE were water-soluble compounds of salvianoli acids including danshensu at 0.51 mg/g, rosmarinic acid at 3.76 mg/g, lithospermic acid at 1.94 mg/g, salvianoli acid A at 1.71 mg/g, and salvianoli acid B at 85.47 mg/g. The contents of these compounds were determined by UPLC-TQ/MS. The RSD of stability and repeatability was below 10%.

### 2.2. Optimization of Mass Spectrometry Conditions

To obtain the best mass spectrometry conditions, the standard solutions of the analytes and internal standard (IS) were separately infused into the mass spectrometer, and were studied in both positive and negative ion modes. The results showed the four analytes and IS had a stronger response in positive ion mode. Product ions were automatically chosen according to the stability and ion response by MS. Declustering potential (DP) and collision energy (CE) for each analyte and IS were also automatically optimized. The optimized MS/MS transitions and MS parameters of four analytes and IS are summarized in Table 1.

### 2.3. Method Validation

#### 2.3.1. Specificity

**Figure 1:**
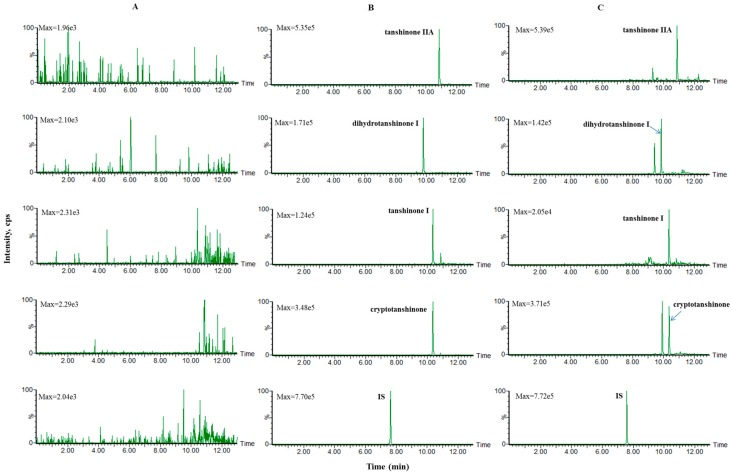
Representative multiple-reaction monitoring chromatograms of (**A**) blank plasma; (**B**) the blank plasma spiked with middle concentration standard solution and IS; (**C**) plasma samples at 2 h after oral administration of SMEE to rats spiked with IS.

Mixed blank plasma samples from six rats were used to investigate the specificity of the method. Representative multiple-reaction monitoring chromatograms of blank plasma, blank plasma spiked with middle concentration standard solution and IS, as well as plasma samples at 2 h after oral administration of SMEE to rats spiked with IS are shown in Figure 1. No endogenous interference from the plasma was observed.

#### 2.3.2. Linearity of Calibration Curves and LLOQs

The calibration curves of four tanshinones exhibited good linearity with correlation coefficients (R^2^) within the range of 0.991–0.999. The calibration curves and LLOQs of the four tanshinones are summarized in Table 2.

#### 2.3.3. Precision

The intra- and inter-day precisions of the four tanshinones in QC samples at three concentration levels are presented in Table 3. The intra-day precision (RSD) ranged from 1.41% to 7.19% and inter-day precision (RSD) ranged from 3.35% to 7.80%.

#### 2.3.4. Matrix Effect and Recovery

The mean matrix effects of the four tanshinones at low, middle, and high concentration ranged from −8.75% to −13.8% RSD ≤ 8.99%. No obvious matrix effect was observed. The mean extraction recoveries of the four tanshinones in plasma at three different concentration levels were found to be from 84.80% to 94.51% with RSD ≤ 7.71%. The data are summarized in Table 4.

#### 2.3.5. Stability

The data of stability experiments are summarized in Table 5, indicating that the four tanshinones in rat plasma were stable after three freeze-thaw cycles and at room temperature for 8 h.

### 2.4. Determination of Biochemical Indicators in Adenine-Induced CRF Models

**Figure 2:**
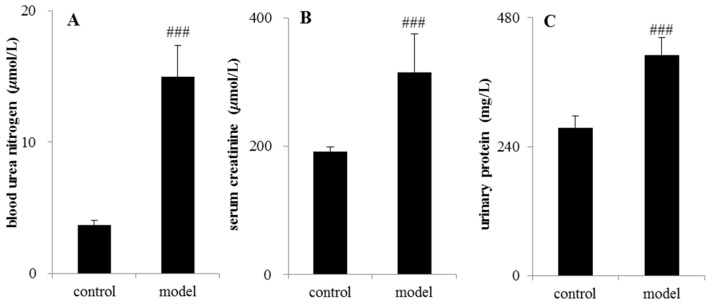
Determination of biochemical indicators between the control group and adenine-induced chronic renal failure group. (**A**) Blood urea nitrogen; (**B**) serum creatinine (Scr); (**C**) urinary protein (UP). ^###^ *p* < 0.001.

The analytical results of biochemical indicators were presented in Figure 2. After 14 days of testing, serum creatinine (Scr), blood urea nitrogen (BUN), and urinary protein (UP) in the CRF rats increased significantly compared with control group (*p* < 0.05 or *p* < 0.01 or *p* < 0.001).

### 2.5. Pharmacokinetics Study

**Figure 3:**
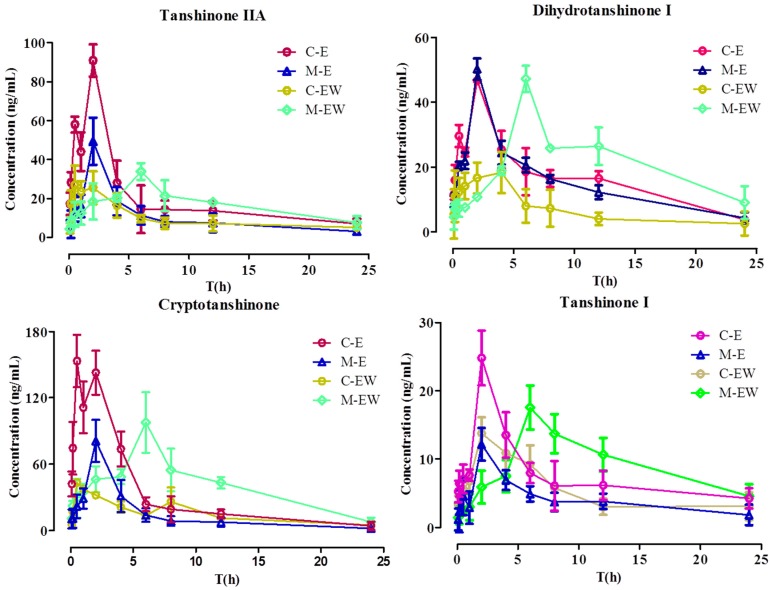
The profiles of the mean plasma concentration versus time of four tanshinones (*n* = 6). C-E: Intragastric administration *Salvia miltiorrhiza* ethanol extract to normal rats; M-E: Intragastric administration *Salvia miltiorrhiza* ethanol extract to CRF rats; C-EW: Intragastric administration *Salvia miltiorrhiza* ethanol extract and water extract to normal rats; M-EW: Intragastric administration *Salvia miltiorrhiza* ethanol extract and water extract to CRF rats.

The validated UPLC-MS/MS method was successfully applied to the pharmacokinetic studies of normal rats administered SMEE or SMEE and SMWE compared with CRF rats (*n* = 6). The profiles of the mean plasma concentration over time for the four tanshinones are illustrated in Figure 3. The main pharmacokinetic parameters calculated by a non-compartment model are presented in Table 6. This was the first report on the pharmacokinetic parameters of four tanshinones in CRF rats and this was also the first report about the influence of SMWE on the pharmacokinetic profile of four tanshinones in CRF rats.

As shown in Table 6, the four tanshinones achieved their maximum plasma concentrations in about 2 h in M-E (intragastric administration *Salvia miltiorrhiza* ethanol extract to CRF rats) group and do not show significant difference from C-E (Intragastric administration *Salvia miltiorrhiza* ethanol extract to normal rats) group except for cryptotanshinone, the T_max_ of which was delayed by about 1.5 h in the M-E group. However, the value of C_max_ and AUC_0–t_ of four tanshinones except for dihydrotanshinone I reduced by 50%~80% and the CLz/F increased by two to four times significantly (*p* < 0.05) in the M-E group, indicating absorption of tanshinone IIA, cryptotanshinone, and tanshinone I were decreased in CRF rats.

After intragastric administration SMWE to C-E group rats, the value of C_max_ and AUC_0–t_ of tanshinone IIA separately decreased from 79.34 μg/L and 534.97 μg/L to 16.61 μg/L and 189.11 μg/L, with CLz/F augmenting from 54.39 μg/L from to 132.76 μg/L, and the variation tendency of the other three tanshinones were the same (*p* < 0.05), which was consistent with our previous reports about the pharmacokinetic influences of polyphenolics on tanshinones [31]. Nevertheless, after intragastric administration SMWE to M-E group rats, the value of C_max_, T_max_, AUC_0–t_ of four tanshinones was upregulation and that of CLz/F was downregulation, which undulated similarity from the model group to the normal group after intragastric administration with compatibility of SMEE and SMWE. These results hinted that SMWE could improve the bioavailability and absorption of tanshinones in CRF rats, but have a converse effect in normal rats.

Pharmacokinetic processes of drugs in the body are divided into absorption, distribution, metabolism, and excretion. Any changes in the link may cause changes in pharmacokinetic parameters. Previous reports also showed that polyphenolics in *Salvia miltiorrhiza* can influence the pharmacokinetic of tanshinones in normal rats [28,29,30], which may be involved in the effect of polyphenolics on blood–brain barrier [32]. Due to the great role of intestinal absorption after intragastric administration and the influence of chronic kidney disease on the structure of gut microbiology [33], the difference of pharmacokinetic parameters of tanshinones in normal and CRF rats may be closely associated with the change of relative enzymes or intestinal bacteria participating in the metabolism, which will be explored by us in the future.

## 3. Materials and Methods

### 3.1. Instruments and Chemicals

Waters Acquity™ Ultra Performance LC system (Waters, New York, NY, USA) equipped with a Quattro Micro MS spectrometer and a Waters Xevo™ G2 TQ MS (Waters MS Technologies, Manchester, UK). MassLynx v4.1 workstation was adopted to analyze the data. Ultrapure water meter (Nanjing Pu Yi Yida Science and Technology Development Co., Ltd., Nanjing, China). Milli-Q system was from Millipore, Bedford, MA, USA. Vacuum freeze-drying equipment (Labconco, UK) and an ultra-high speed centrifuge at low temperature (Thermo, Renfrew, UK) were used. UPLC-grade acetonitrile and formic acid were purchased from Sigma-Aldrich (St. Louis, MO, USA). Standard substance Dihydrotanshinone I (20140516) was bought from Nanjing spring autumn biological engineering Co., Ltd. (Nanjing, China). Tanshinone I (110867–200406), Tanshinone IIA (110766–200619) and Clarithromycin (Internal standard, 130356–200403) were purchased from National Institute for Food and Drug Control (Beijing, China). Cryptotanshinone (110852–200806) were obtained from National Institute for the Control of Pharmaceutical and Biological Products (Beijing, China). *Salvia miltiorrhizae* Radix et Rhizoma (20150701) was bought from Bozhou city traditional Chinese medicinal materials market.

### 3.2. Preparation and Determination of SMEE and SMWE

The air-dried *Salvia miltiorrhizae* Radix et Rhizoma (400 g) was extracted with 2 L purified water three times with ultrasound wave extract at ambient temperature. The extraction was concentrated to 1 g/mL and precipitated with 80% ethanol for 24 h at 4 °C. The supernatant was concentrated and freeze dried into powder as SMWE (62 g). The residue after water extraction was extracted again with 2 L 95% ethanol three times in the same way. After being concentrated under reduced pressure, it was freeze dried into powder as SMEE (26 g). SMEE (40 mg), and SMWE (50 mg) were separately accurately weighed and dissolved in 10 mL 90% methanol containing IS with the concentration of 136.56 ng/mL. The solution was centrifuged at 13,000 rpm for 10 min and 5 μL of the supernatant was injected for UPLC-TQ/MS analysis.

### 3.3. UPLC-TQ/MS Condition

The UPLC-TQ/MS condition refers the previous report [31] with some modifications. The quantitative analysis of the chemical constituents was carried out on Thermo Syncronis C_18_ (100 mm × 2.1 mm, 1.7 μm) by UPLC-TQ/MS. A gradient mobile phase system was composed by water (containing 0.1% HCOOH) (A)-acetonitrile (B) at a flow rate of 0.4 mL·min^−1^. The optimized UPLC elution conditions were: 0~1 min, 3%~3% B; 1~6 min, 3%~30% B; 6~7 min, 30%~40% B; 7~10 min, 40%~95% B, 10~12 min, 95% B, 12~12.2 min, 95%~3% B. The sample injection was 5 μL and the column temperature was maintained at 35 °C. The autosampler was maintained at 4 °C.

The mass spectrometer was operated in the positive multiple-reaction monitoring mode. The capillary and cone voltage were set at 3.0 kV and 30 V, respectively. The desolvation gas was set to 1000 L/h at a temperature of 500 °C, the cone gas was set to 50 L/h and the source temperature was set to 150 °C. The data acquisition rate was set to 0.2 s. All of the data acquisition and analyses of data were controlled by Waters MassLynx v4.1 software.

### 3.4. Standard Solutions Preparation

The appropriate amounts of four tanshinones were separately weighed and dissolved in methanol as the stock solutions. Mixture stock solution was prepared by mixing and diluting the above-mentioned four stock solutions with methanol to achieve final concentrations of 108 μg/mL for tanshinone IIA, 114 μg/mL for dihydrotanshinone I, 107 μg/mL for tanshinone I, and 111 μg/mL for cryptotanshinone. The mixture stock solution was serially diluted with methanol at ratios of 1:100, 1:200, 1:1000, 1:2000, 1:10,000, 1:20,000, and 1:100,000 to provide a series of working standard solutions at appropriate concentrations in the range of 0.00108–1.08 μg/mL for tanshinone IIA, 0.00114–1.14 μg/mL for dihydrotanshinone I, 0.00107–1.07 μg/mL for tanshinone I, 0.001 and 11–1.11 μg/mL for cryptotanshinone. These series of working standard solutions after addition IS with the final concentrations of 136.56 ng/mL drew a curve, which was applied to calculate the content of four tanshinones and five water soluble salvianolic acids in SMEE and SMWE.

The plasma samples at 24 h from C-E, M-E, C-EW, and M-EW group rats were detected and the concentration were 4.39, 3.69, 3.58, 4.08 ng/mL for tanshinone IIA; 4.26, 4.09, 3.81, 3.94 ng/mL for tanshinone I; 4.52, 4.33, 3.88, 4.26 ng/mL for dihydrotanshinone I; and 4.15, 3.29, 3.82, 4.48 ng/mL for cryptotanshinone, respectively. The plasma concentration at 24 h of four tanshinones was close to 4 ng/mL, so the low concentration of quality control (QC) standard work solutions of four tanshinones was set near to 4 ng/mL. Hence, high, middle, and low concentrations of QC standard work solutions were prepared by diluting the highest concentration point of the calibration curve according to the ratios of 1:1, 1:5, and 1:25, respectively. Three concentrations of QC work solutions were 108, 21.6, 4.32 ng/mL for tanshinone IIA; 114, 22.8, 4.56 ng/mL for dihydrotanshinone I; 107, 21.4, 4.28 ng/mL for tanshinone I; and 111, 22.2, 4.44 ng/mL for cryptotanshinone. IS working solution (136.56 ng/mL) was prepared by diluting the stock solution (122.5 μg/mL) with methanol. All the solutions were stored at −20 °C.

### 3.5. Sample Preparation

The plasma samples were prepared by one-step direct protein precipitation with three times the volume of methanol.

The calibration curve samples and QC samples were prepared by separately adding 20 μL of a series of working standard solutions or 20 μL of three concentrations of standard work solutions, to 50 μL of blank plasma, then 130 μL of IS solution was spiked. Samples of rat plasma (50 μL) were mixed with 20 μL of methanol and 130 μL of IS solution. After vortex-mixing for 30 s and centrifugation at 13,000 rpm for 10 min at 4 °C, 5 μL of the supernatant was injected for UPLC-TQ/MS analysis.

### 3.6. Method Validation

#### 3.6.1. Specificity

Blank plasma samples from different rats were prepared and analyzed to eliminate the interferences from endogenous compounds. IS addition to blank sample with analytes was analyzed to investigate the potential interferences of IS. The chromatograms from blank plasma, blank plasma spiked with IS, blank plasma spiked with analytes and IS, as well as plasma samples after oral administration of SMEE were compared.

#### 3.6.2. Linearity of Calibration Curves and Lower Limits of Quantification (LLOQ)

The calibration curves for these four compounds were formed by plotting peak-area ratios (Y) of each analyte to the IS versus plasma concentrations (X), using the least-square linear regression with a weighting factor 1/X. The ratio of signal to noise above 10 was defined as LLOQ.

#### 3.6.3. Precision

The intra-day and inter-day precision of four tanshinones were separately estimated by determining six replicate QC samples at three different concentration (low, medium, and high) levels on the same day and on three consecutive days using calibration curves obtained daily. RSD of the precision must be below 15% at each concentration level [34].

#### 3.6.4. Extraction Recovery and Matrix Effect

The extraction recovery and matrix effect of four tanshinones were determined by analyzing six replicates of plasma samples at three QC concentration levels. The extraction recoveries evaluated by comparing the content of the 20 μL analytes spiked in 50 μL blank plasma precipitated with 130 μL IS calculated by standard curve with the actual additive content. The matrix effect was calculated by comparing the peak areas of the 20 μL analytes spiked in 50 μL blank plasma precipitated with 130 μL IS with those spiked in 50 μL water addition to 130 μL IS.

#### 3.6.5. Stability

The stability of all analytes in rat plasma was studied by analyzing six replicate QC samples at three different concentrations including: room-temperature stability, freeze, and thaw stability.

The freeze and thaw stability were determined after three freeze-thaw cycles (from −80 °C to 25 °C). The room-temperature stability was assessed by analyzing QC samples kept at room temperature for 8 h. All QC samples were determined by using a daily calibration curve. The RSD of stability by calculating the concentration should be within ±15% [34].

#### 3.6.6. Animals

All experimental procedures were carried out in accordance with the Guide for the Care and Use of Laboratory Animals, and before the animal experiments were carried out, the procedures were approved by the Research Ethics Committee of Nanjing University of Chinese Medicine (Nanjing, China). Male SD rats were obtained from the Central Animal Breeding House of Nanjing University of Chinese Medicine (Nanjing, China). They were housed in cages with a constant humidity (ca. 60% ± 2%) and temperature (ca. 25 ± 1 °C) with a light/dark cycle of 12 h. The animals were used for six weeks and underwent an adaptation period of several days, during which they were given free access to water and food.

The 24 rats weighing 190~210 g were randomly divided into two groups (*N* = 12/group), a control group and model group, after measuring their body weight. The model group was given 150 mg/kg·d body weight of adenine dissolved with 1% (*w*/*v*) gum acacia solution (by gastric gavage) for 14 days to establish an adenine-induced CRF model. Urine and plasma were collected for biochemical indicator measurements—Scr, BUN, and UP—which were applied to evaluate the model. During those 14 days, the control group was similarly given an equal volume of gum acacia solution every day. Body weight was measured daily for all rats. On the last day, the control rats were divided into C-E group and C-EW group. The model rats were also divided into an M-E group and M-EW group. Both C-E and M-E groups were gavaged 0.65 g/kg SMEE (equivalent to 16.42 mg/kg of tanshinone IIA, 13.36 mg/kg of dihydrotanshinone I, 56.85 mg/kg of cryptotanshinone, 8.12 mg/kg of drotanshinone I). Both C-WE and M-WE groups were administered 0.65 g/kg of SMEE combined with 1.55 g/kg SMWE. Blood samples were collected in 1.5 mL heparinized tubes via the postorbital venous plexus veins from each rat before drug administration and at 0.083, 0.17, 0.5, 1, 2, 4, 6, 8, 12, and 24 h after drug administration. The blood samples were centrifuged at 3000 rpm for 10 min. The plasma was collected and stored at −80 °C until analysis.

#### 3.6.7. Applications in Pharmacokinetic Studies

The pharmacokinetic analysis was performed by a non-compartmental approach using the DAS version 3.0 (BioGuider Co., Shanghai, China). The following pharmacokinetic parameters were calculated: area under the plasma concentration–time curve from time zero to infinity (AUC_0–∞_), area under the plasma concentration–time curve from zero to the time of last measurable concentration (AUC_0–t_), maximum observed plasma concentration (C_max_), time to first occurrence of C_max_ (t_max_), half-life in elimination phase (t_1/2_), volume of distribution (Vz/F), mean residence time (MRT).

## 4. Conclusions

In this study, a simple, rapid, and sensitive UPLC-TQ/MS method was successfully established and applied to determine four tanshinones in SMEE before and after combination with SMWE simultaneously in normal and CRF rats. The pharmacokinetic profile of the four tanshinones were altered in CRF rats and also varied after oral administration with compatibility of SMEE and SMWE. The results indicated that four tanshinones have slower uptake and higher elimination in CRF rats. After administering the SMWE and SMEE combination, absorption increases and elimination slows down in CRF rats. The results provide scientific information for further exploration of the mechanisms of the combination of SMWE and SMEE and offer a reference for clinical administration of SM.

## Figures and Tables

**Table 1:** The retention time (t_R_), optimized MS/MS transitions, declustering potential (DP) and collision energy (CE) for four analytes and IS.

Analytes	t_R_ (min)	Precursor Ion (*m*/*z*)	Product Ion (*m*/*z*)	DP (V)	CE (eV)
Tanshinone IIA	10.87	295.1	190.8	32	44
Dihydrotanshinone I	9.80	279.2	189.9	28	34
Cryptotanshinone	10.34	297.1	251.1	36	26
Tanshinone I	10.36	277.2	178.0	30	36
Clarithromycin (IS)	7.56	748.6	82.9	24	46

**Table 2:** The regression equations, linear ranges, correlation coefficient (R^2^), and LLOQs for four tanshinones in rat plasma (*n* = 6).

Analytes	Regression Equation	R^2^	Linearity Range (ng/mL)	LLOQ (ng/mL)	CV ‰ of Calibration Slopes
Tanshinone IIA	*y* = 0.0195*x* + 0.0258	0.9990	0.540–108	0.540	0.500
Dihydrotanshinone I	*y* = 0.0119*x* + 0.0073	0.9993	0.570–114	0.570	0.350
Cryptotanshinone	*y* = 0.0224*x* + 0.0317	0.9993	0.555–111	0.555	0.350
Tanshinone I	*y* = 0.0107*x* + 0.0305	0.9974	0.535–107	0.535	1.301

**Table 3:** The intra- and inter-day precisions of four tanshinones in QC samples at three concentration levels (*n* = 6 replicates per day).

Analytes	Spiked Conc. (ng/mL)	Intra-Day		Inter-Day	
		Measured Conc. (ng/mL)	RSD (%)	Measured Conc. (ng/mL)	RSD (%)
Tanshinone IIA	4.32	4.07 ± 0.07	1.75	4.09 ± 0.14	3.49
	21.6	19.14 ± 0.57	3.00	19.57 ± 0.67	3.40
	108	90.75 ± 1.27	1.41	91.95 ± 4.32	4.70
Cryptotanshinone	4.44	3.89 ± 0.23	6.01	3.93 ± 0.31	7.80
	22.2	19.93 ± 1.17	5.87	20.52 ± 1.17	5.69
	111	89.45 ± 2.03	2.20	94.20 ± 1.15	3.35
Dihydrotanshinone I	4.56	4.23 ± 0.30	7.19	4.22 ± 0.25	5.93
	22.8	20.82 ± 0.44	2.12	20.91 ± 1.14	5.46
	114	99.7 ± 4.37	4.38	97.8 ± 3.72	3.80
Tanshinone I	4.28	4.01 ± 0.20	4.92	4.00 ± 0.18	4.63
	21.4	19.87 ± 0.75	3.78	19.82 ± 0.85	4.29
	107	94.88 ± 3.77	3.98	97.57 ± 3.94	4.03

**Table 4:** Extraction recovery and matrix effects of four tanshinones in QC samples at three concentration levels (*n* = 6).

Analytes	Spiked Conc. (ng/mL)	Recovery (%)	RSD (%)	Matrix Effects (%)	RSD (%)
Tanshinone IIA	4.32	93.19 ± 3.56	3.82	−13.8	8.99
	21.6	90.35 ± 2.46	2.72	−10.12	5.71
	108	84.80 ± 3.57	6.45	−12.83	7.89
Cryptotanshinone	4.44	89.98 ± 6.93	7.71	−12.08	7.52
	22.2	92.72 ± 4.67	5.03	−8.75	6.52
	111	86.52 ± 2.79	3.22	−11.23	6.58
Dihydrotanshinone I	4.56	92.17 ± 3.05	3.30	−12.8	7.46
	22.8	89.51 ± 5.14	5.74	10.88	6.34
	114	86.28 ± 2.79	3.23	8.64	6.13
Tanshinone I	4.28	94.16 ± 2.29	2.43	11.68	6.68
	21.4	94.51 ± 4.01	4.24	7.05	5.80
	107	91.02 ± 3.77	4.14	14.34	8.35

**Table 5:** Room-temperature stability and triple freeze-thaw stability of four tanshinones in QC samples at three concentration levels (*n* = 6).

Analytes	Spiked Conc. (ng/mL)	Room-Temperature Stability		Freeze-Thaw Stability	
		Measured Conc. (ng/mL)	RSD (%)	Measured Conc. (ng/mL)	RSD (%)
Tanshinone IIA	4.32	4.05 ± 0.24	5.98	4.11 ± 0.24	5.77
	21.6	19.58 ± 0.57	2.90	19.62 ± 0.37	1.83
	108	90.39 ± 4.07	4.51	92.15 ± 4.40	4.77
Cryptotanshinone	4.44	3.91 ± 0.30	7.56	3.86 ± 0.24	6.20
	22.2	20.47 ± 0.86	4.21	20.27 ± 1.17	5.75
	111	96.75 ± 4.09	4.22	96.55 ± 3.31	3.43
Dihydrotanshinone I	4.56	4.12 ± 0.18	4.32	4.1 ± 0.16	3.79
	22.8	20.4 ± 0.88	4.35	19.67 ± 1.48	1.89
	114	96.79 ± 2.51	2.59	94.75 ± 3.25	3.43
Tanshinone I	4.28	3.9 ± 0.24	6.13	3.91 ± 0.24	5.41
	21.4	20.05 ± 0.89	4.44	20.24 ± 0.89	3.69
	107	92.98 ± 3.73	4.01	91.54 ± 3.73	2.56

**Table 6:** Pharmacokinetics parameters of the four tanshinones after intragastric administration of SMEE or SMEE and SMWE (mean ± SD, *n* = 6). C-E: Intragastric administration *Salvia miltiorrhiza* ethanol extract to normal rats; M-E: Intragastric administration *Salvia miltiorrhiza* ethanol extract to CRF rats; C-EW: Intragastric administration *Salvia miltiorrhiza* ethanol extract and water extract to normal rats; M-EW: Intragastric administration *Salvia miltiorrhiza* ethanol extract and water extract to CRF rats. * *p* < 0.05, ** *p* < 0.01, *** *p* < 0.001: M-E vs. C-E; **^#^** *p* < 0.05, **^##^** *p* < 0.01, **^###^** *p* < 0.001: C-EW vs. C-E; ^+^ *p* < 0.05, ^++^ *p* < 0.01, ^+++^ *p* < 0.001: M-EW vs. M-E; ^∆^ *p* < 0.05, ^∆∆^ *p* < 0.01, ^∆∆∆^ *p* < 0.001: M-EW vs. C-EW.

Parameters		Tanshinone IIA	Cryptotanshinone	Dihydrotanshinone I	Tanshinone I
C_max_ (μg/L)	C-E	79.341 ± 13.96	156.57 ± 6.34	45.66 ± 1.68	25.54 ± 1.27
	M-E	25.46 ± 8.44 ^***^	47.64 ± 14.34 ^***^	50.29 ± 24.78	11.22 ± 0.97 ^***^
	C-EW	16.61 ± 1.10 ^###^	44.32 ± 4.66 ^###^	14.46 ± 3.42 ^###^	12.29 ± 1.29 ^###^
	M-EW	31.10 ± 3.64 ^+,∆∆∆^	94.02 ± 12.89 ^+++,∆∆^	49.17 ± 24.37 ^∆∆^	16.55 ± 0.94 ^+++,∆∆^
T_max_ (h)	C-E	1.17 ± 0.76	0.67 ± 0.29	2.00	1.75 ± 0.5
	M-E	2.00	2.00 ^***^	2.00	2.00
	C-EW	0.50	0.50	2.00	2.00
	M-EW	7.33 ± 1.16 ^+++,∆∆∆^	7.33 ± 1.56 ^+++,∆∆∆^	6.67 ± 1.16 ^+++,∆∆∆^	6.00 ^+++,∆∆∆^
T1/2 (h)	C-E	10.218 ± 5.43	5.83 ± 2.54	7.63 ± 0.29	16.15 ± 5.78
	M-E	9.42 ± 0.69	5.49 ± 2.05	8.31 ± 2.33	10.51 ± 2.80 ^*^
	C-EW	21.03 ± 0.83 ^##^	8.20 ± 2.08 ^#^	11.81 ± 1.39 ^##^	4.92 ± 2.17 ^##^
	M-EW	7.14 ± 3.00 ^∆∆∆^	3.96 ± 0.98 ^∆∆^	6.83 ± 1.90 ^∆∆∆^	11.59 ± 3.63 ^∆∆^
AUC_0–t_ (μg·h/L)	C-E	534.968 ± 139.63	1036.80 ± 203.01	432.46 ± 53.53	222.23 ± 56.62
	M-E	236.93 ± 58.16 ^**^	283.44 ± 131.47 ^***^	350.24 ± 9.31 ^**^	88.05 ± 15.81 ^***^
	C-EW	189.11 ± 1.05 ^###^	324.95 ± 17.25 ^###^	158.90 ± 9.86 ^###^	121.73 ± 16.19 ^###^
	M-EW	409.46 ± 4.74 ^++,∆∆∆^	984.76 ± 22.52 ^+++,∆∆∆^	502.33 ± 145.21 ^+,∆∆^	208.89 ± 12.22 ^+++,∆∆∆^
AUC_0–∞_ (μg·h/L)	C-E	633.761 ± 165.01	1069.96 ± 183.93	479.38 ± 57.15	301.17 ± 83.46
	M-E	283.16 ± 60.67 ^**^	297.17 ± 137.96 ^***^	382.88 ± 16.74 ^*^	111.17 ± 30.66 ^**^
	C-EW	339.91 ± 9.52 ^###^	372.06 ± 40.33 ^###^	208.57 ± 3.43 ^###^	128.00 ± 22.18 ^##^
	M-EW	470.04 ± 44.32 ^+,∆∆^	1016.17 ± 18.84 ^+++,∆∆∆^	599.14 ± 220.73 ^+,∆^	292.26 ± 52.21 ^++,∆∆^
MRT_0–t_ h	C-E	7.27 ± 0.26	5.36 ± 0.63	7.86 ± 0.04	9.17 ± 0.23
	M-E	8.02 ± 0.49 ^*^	6.01 ± 0.91	7.44 ± 1.08	8.50 ± 1.00
	C-EW	9.0 ± 1.45 ^#^	7.24 ± 0.81 ^#^	8.87 ± 1.33 ^#^	8.55 ± 0.92
	M-EW	9.17 ± 0.60 ^+^	8.14 ± 0.49 ^+^	10.22 ± 0.88 ^+^	10.68 ± 0.30 ^+,∆^
MRT_0–∞_ h	C-E	12.51 ± 4.38	6.34 ± 0.15	10.53 ± 0.15	18.42 ± 7.10
	M-E	12.97 ± 1.47	7.18 ± 1.95	11.14 ± 3.74	18.02 ± 6.94
	C-EW	22.81 ± 11.75 ^#^	10.83 ± 2.99 ^#^	21.87 ± 10.31 ^#^	9.63 ± 2.23 ^#^
	M-EW	12.49 ± 3.41 ^∆^	8.82 ± 1.00	13.67 ± 3.04 ^∆^	19.23 ± 4.71 ^∆^
CLz/F	C-E	54.39 ± 14.99	108.47 ± 19.36	56.24 ± 6.29	57.00 ± 16.87
	M-E	119.37 ± 23.90 ^***^	440.76 ± 194.22 ^*^	65.09 ± 8.53	128.26 ± 50.49 ^**^
	C-EW	132.76 ± 62.56 ^##^	308.13 ± 35.06 ^##^	106.97 ± 36.68 ^#^	129.61 ± 23.74 ^##^
	M-EW	70.27 ± 6.43 ^+++,∆∆∆^	111.89 ± 0.21 ^++,∆∆∆^	49.39 ± 19.75 ^∆∆^	56.73 ± 9.83 ^++,∆∆^
